# Enhanced low-gamma band power in the hippocampus and prefrontal cortex in a rat model of depression is reversed by orexin-1 receptor antagonism

**DOI:** 10.1016/j.ibneur.2023.11.006

**Published:** 2023-11-22

**Authors:** Batoul Mirbolouk, Behrooz Khakpour-Taleghani, Mohammad Rostampour, Adele Jafari, Kambiz Rohampour

**Affiliations:** a Department of Physiology, School of Medicine, Guilan University of Medical Sciences, Rasht, Iran; b Neuroscience Research Center, Guilan University of Medical Sciences, Rasht, Iran

**Keywords:** Chronic mild stress, Depression, Orexin-1 receptor, Local field potential, Gamma band power, Coherence

## Abstract

The hippocampal-prefrontal cortex network dynamics is reported to be involved in various cognitive functions and in different mood disturbances including depression. It has been suggested that blocking orexin-1 receptors can be beneficial in depression. The purpose of this study is to determine whether orexin-1 receptor antagonists have an impact on changes in brain oscillations in the hippocampus and prefrontal cortex in a rat model of depression. Forty-eight male Wistar rats were divided into six experimental groups: control, chronic mild stress (CMS), acute SB-334867, a selective orexin-1 receptor antagonist, treated rats (SB), chronic SB-treated (CSB), CMS+SB, and CMS+CSB. Two stainless steel recording electrodes were placed in the coordinates of the hippocampus (HPC) and the prefrontal cortex (PFC). After behavioral verification of the model, local field potentials were recorded at 1 kHz sampling frequency. The absolute power of different frequency bands was obtained using the Fast Fourier Transform (FFT) function, and the power spectral density (PSD) of each frequency band was calculated for each animal. In the CMS- treated animals, the low-gamma band power increased both in the HPC and PFC (p ≤ 0.05), which were reversed by chronic SB-334867 treatment (p ≤ 0.05). The alterations in theta, and high-gamma band power were not significant in CMS treated rats, while acute and chronic SB-334867 treatment diminished the theta and high-gamma band power (p ≤ 0.05), respectively. The hippocampal-prefrontal coherence decreased in the delta (p ≤ 0.01), theta (p ≤ 0.01), and alpha (p ≤ 0.05) band range of the CMS exposed rats. It is concluded that CMS boosts the low-gamma band power, which is reversed by CSB treatment. The low-frequency band coherence is attenuated after CMS treatment.

## Highlights

• Low-gamma band power increased in the hippocampus of animal models of depression.
• Blocking the orexin-1 receptor prevented the gamma potentiation, in depressed rats.
• Hippocampal–prefrontal coherence decreased in depressed rats.

## 1 Introduction

Over 300 million individuals, i.e., 4.4% of the world's population, are affected by the common mental condition known as depression. Depression also leads to 7.5% of living with permanent disability in the affected population (WHO, 2017). Despite the growing scientific knowledge about this destructive disease, its biological reason is still poorly understood. Many brain regions including the prefrontal cortex (PFC), and the hippocampus (HPC), are influenced by depression. Physiological variation in the PFC can be important since it is essential for the regulation of stress responses Arnsten A,F (2015) There is evidence suggesting depressed patients exhibit volume loss in their hippocampus and prefrontal cortex. Furthermore, postmortem examination of tissue from the depressed patient reveals decreased neuron number and size, reduced synaptic interaction, alterations in their protein expression, and reductions in trophic factor expression (Karege, 2005). Exposure to stress is one of the key risk factors for the formation and growth of depressive symptoms in people (Hettema, 2006). One of the methodological approaches to assess the behavioral variation in the prevalent animal models is chronic stress experimental. These variations comprise changed neuronal structure and loss of trophic factor support (Duman, R.S., 2006). There is proof that the enhancement of trophic factor signaling and subsequent synaptogenesis inside the PFC constitute a crucial part of the positive effects of rapid-acting antidepressants Autry et al., (2011). Recent research indicates that both typical and rapid-acting antidepressants directly bind to the TrkB receptor and allosterically potentiating brain-derived neurotrophic factor (BDNF) signaling (Casarotto, 2022), which is a key regulator of synaptic plasticity (Leal, 2017).

The orexin neuropeptides are synthesized exclusively in the hypothalamus. However, it regulates all brain activity globally and regulates a variety of complex behaviors such as food intake, sleep/wake, reward, and emotion (Zhang, 2013). There are two orexin neuropeptides, orexin A (OXA) and orexin B (OXB), which are expressed only in a small group of neurons in the lateral region of the hypothalamus (Sakurai, 1998). Brundin et al. reported a negative relationship between major depressive disorder (MDD) and orexin-A levels Brundin et al., (2007). While others report positive (von der Goltz et al., 2011), or no related link between orexin-A and MDD (Schmidt, 2010).

Orexins' critical roles in the activation and regulation of the HPA axis are demonstrated by new evidence. Abnormal stress reactivity can result from orexin system disturbance Akça et al., (2020). According to a number of studies conducted on animals, orexin appears to activate the HPA axis by causing the hypothalamus and adrenal glands to produce glucocorticoids and corticotropin-releasing hormone, respectively Blasiak et al., (2015). The hippocampus and medial PFC (mPFC), which are critical in controlling reactions related to stress and anxiety, express orexin terminals (Johnson, 2012). Furthermore, orexinergic neurons transmit fibers to the nucleus accumbens and the ventral tegmental area (VTA) (Sakurai, 2014).

The hippocampal-prefrontal cortex circuit is involved in various cognitive functions, including memory formation, spatial navigation, and executive function (Eichenbaum, 2007). Disruptions to this circuit can affect mental health, with research suggesting a potential role in conditions such as post-traumatic stress disorder (PTSD) (Bremner, 2003), and depression (Godsil, 2013). Studies show that cognitive and memory deficits in depression are due to the inhibition of synaptic plasticity in the mPFC (Quan, 2011). The electroencephalogram (EEG) of individuals with MDD shows a higher absolute or relative alpha band power (Prichep, 1992) primarily at parietal and frontal sites (Grin-Yatsenko et al., 2009, Jaworska et al., 2012). According to other reports, the rostral anterior cingulate cortex (rACC), has been linked to theta band abnormalities in depressive patients. A strong correlation between high rACC activity and decreased Hamilton Rating Scale of Depression (HAM-D) scores were revealed (Pizzagalli, 2018). In the present study, we evaluated the alterations in power spectral density (PSD), and coherence of the hippocampus and the PFC in a rat model of depression which was treated with an orexin-1 receptor antagonist, SB-334867.

## 2 Method and material

### 2.1 Animals

**Fig. 1:**
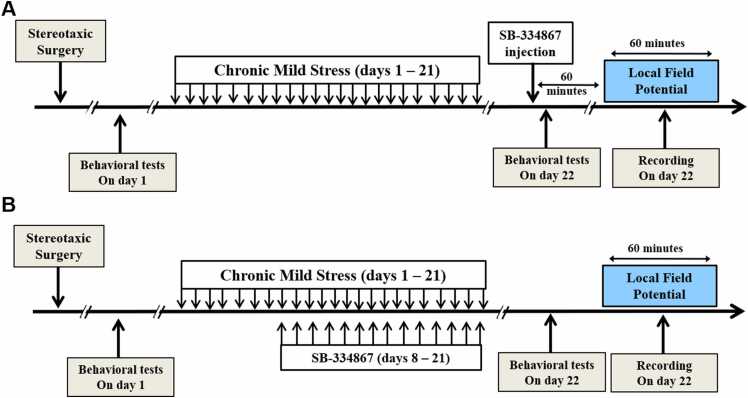
Timeline of the experimental procedure. Acute administration of SB-334867 was performed on day 22 immediately before the behavioral tests, which was one hour before the LFP recording (A). For the chronic treatment with SB-334867, it was applied daily for 14 days (B).

A total of 48 male Wistar rats weighing 250–320 g was provided by the animal house of the Faculty of Medicine, Guilan University of Medical Sciences. All animals were kept at 22 ± 2 °C and randomly assigned to one of six experimental groups: control, chronic mild stress (CMS), acute SB-treated (SB), chronic SB-treated (CSB), CMS+SB, and CMS+CSB. Rats had access to food and water ad libitum. Animals with acute SB treatment received a single intracerebroventricular (icv) injection on day 22, immediately before the behavioral tests, and 60 min before the local field potential recordings. Chronic SB treatment was performed for two weeks (day 8 – 21). Fig. 1 shows the timeline of the experiment.

### 2.2 Stereotaxic surgery

The rats were anaesthetized with intraperitoneal (i.p.) injections of ketamine (80 mg/kg) and xylazine (20 mg/kg). After shaving, the head was placed in the stereotaxic instrument, and the scalp was disinfected with 70% alcohol. A guide cannula made by a 22-gauge needle was implanted in the coordination of the lateral ventricle according to the rat brain atlas of Paxinos: AP = 0, DV = 4, L = 1.5 relative to the Bregma. Afterwards, the stereotaxic instrument was set in the coordinates of the prefrontal cortex (AP = +3.3, DV = 3.3, and L = 0.7), and the hippocampus area at the coordinates AP = −3.3, DV = 3.6, and L = 3.3, and the coordination of the HPC and the PFC were marked for the recoding time (in 3 weeks). The guide cannula was fixed to the skull by dental cement and two screws. A stylet was inserted into the cannula to prevent its blockage. A small amount of tetracycline powder was poured on the surface of the skull to prevent infection. Then, the skin of the head was sutured, and the rats underwent a recovery period for one week.

### 2.3 The chronic mild stress induction protocol

The chronic mild stress (CMS) induction protocol began one week after surgery as described elsewhere (Mirbolouk, 2023). Briefly, the animals were unexpectedly exposed to various stresses over three weeks. Every day, the animals were exposed to at least one stressor. These stressors included enduring exposure to white noise (80 dB) for 12 h, being deprived of food or water for 18 h, staying in wet cages for 21 h, being in cold water for 6 min, tilting the cage for 10 h, being physically restrained for 2 h, and being exposed to continuous illumination for 36 h.

### 2.4 Behavioral verification of CMS induction

Rats were made to spend five minutes in a water-filled, cylinder-shaped piece of plexiglass during a forced swim test (FST). In order to accommodate the animal with the test environment, 24 h before the FST rats were placed in the water filled cylinder for 15 min. The animal's total immobility time in the test session was tracked and analyzed. This is the amount of time (in seconds) the animal did not attempt to leave the cylinder and only made minimal movements to avoid drowning. The immobility time is considered as an index of depressive-like behavior.

The rats were placed in an open field chamber whose floor was divided into 16 tiny partitions with a permanent marker in order to measure their locomotor activity. During the five minutes of exploration, the number of crosses made over each divider was assessed as an anxious indicator.

In order to assess the anhedonia-like symptoms the sucrose preference test (SPT) was performed, rats had a 48-hour adaptation phase, three days prior to the SPT, during which they were introduced to two bottles containing 2% sucrose. The animals were dehydrated for 20 h before the test of sucrose preference (Bosch, 2022). They were then exposed for three hours to two bottles, one containing only tap water and the other containing water sweetened with 2% sucrose. After weighing the bottles at the end of the assessment period, the percentage of sucrose preference for each rat was calculated as follows: (weight of water consumed from the sucrose-containing bottle) divided by (weight of the total amount of water consumed from both bottles) times 100.

All behavioral tests were performed on the same day (day 22) between 8 and 12 AM (in the inactive phase), with 15 min intervals between different tests.

### 2.5 Local field potential recording

**Fig. 2:**
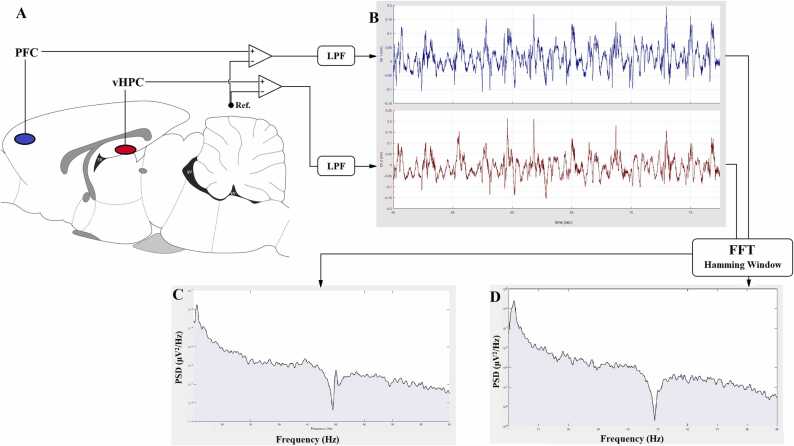
**-** Schematic diagram showing the experimental setup of the LFP recording. Signals retrieved from HPC and PFC were relayed to a differential amplifier, and compared with the voltage of the reference electrode placed on the skull (A). Sample LFP recordings are shown in B, which were analyzed with Fast Fourier Transform (FFT) and the individual PSD traces (C and D) were statistically analyzed.

Rats were anaesthetized with urethane (1.5 g/Kg) following the behavioural testing and model validation. The scalp was carefully dissected, the previously marked locations were thoroughly drilled with a dental drill, and two electrodes were positioned unilaterally at the depth of the aforementioned coordinates. Additionally, a screw with a connection for the reference electrode was positioned on the skull. The recording electrodes were mounted via an electrode holder and attached to the headstage of the BioDAQ data acquisition system (Trita Inc., Iran). The recording electrodes were made up of two strands of PFA (Perfluoroalkoxy)-coated stainless steel wire (A-M Systems). A sampling frequency of 1 kHz was used to record the brain signals after they had been thousand times amplified. A band-pass filter of 0.1–100 Hz and a 50 cycle Notch filter were applied. Fig. 2A shows the experimental setup of the LFP recording, and the sample LFP signals are presented in Fig. 2B.

### 2.6 Data analysis

Ten-minute epochs of the LFP recording were used for offline analysis in the MATLAB software environment (MathWorks, Inc., USA). The absolute power of different frequency bands (delta: 0.5–4 Hz, theta: 4–8 Hz, alpha: 8–13, beta: 13–30 Hz, low gamma: 30–50 Hz, and high gamma: 50–80 Hz) were obtained using the Fast Fourier Transform (FFT) function. The power spectral density (PSD) of each frequency band was calculated for each animal, using the *pwelch* method. A hamming window with 50% overlap was used for the analysis of PSD. Overall coherence between the hippocampus and the PFC was calculated for each frequency band, using the *mscohere* function in the Matlab environment.

The Graphpad Prism software was used to statistically analyze the final results. To determine if the data had a normal distribution, the Shapiro-Wilk test was applied. One-way analysis of variance (ANOVA) was used to determine the significance of PSD data with normal distributions, and Tukey post-hoc analysis was used for pairwise comparisons. Otherwise, data were analyzed using the Kruskal-Wallis test. The Bonferroni correction was used for multiple comparisons in the power spectrum analysis. An independent t-test was used to compare the coherence of HPC and PFC oscillations in CMS-treated and control rats. The presentation of all data is as Mean ± SEM, with P < 0.05 being considered significant.

## 3 Results

### 3.1 Behavioral confirmation of the CMS model

**Fig. 3:**
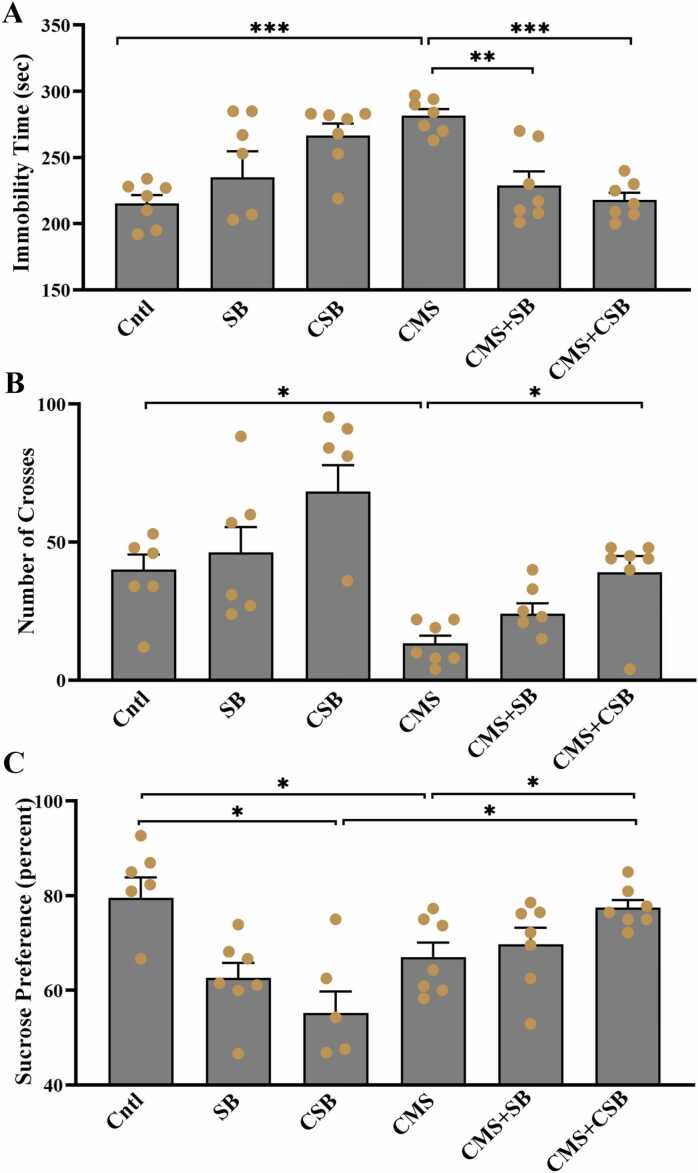
**-** Alterations in behavioral symptoms observed in different groups after three weeks of treatment. Immobility time in the forced swim test (A), Number of crosses in the open field test (B), and percentage of sucrose consumption in the sucrose preference test (C) Data are shown as Mean ± SEM (One-way ANOVA, * p ≤ 0.05, ** p ≤ 0.01, *** p ≤ 0.001, n = 7).

The behavioral results of the anti-depressive effects of SB-334867 were previously reported in detail (Mirbolouk, 2023); Fig. 3 indicates the induction of depressive-like, and anxiety-like behaviors (LFP) in the CMS model before LFP recordings. Despite the fact that 48 rats (n = 8) were initially included in the trial, some of the animals were removed because they forced out of their connection sockets during the CMS procedure. As a result, the total number of data points may vary in each experimental group. The behavioral results indicate that three weeks of exposure to CMS induced different depressive-like behaviors. The one-way ANOVA indicated, the immobility time in SPT increased significantly (F (5, 36) = 6.576, P = 0.0002) from 216.4 ± 8.3 s in intact animals to 279.8 ± 5.5 s, in the CMS group. While both acute (p ≤ 0.01) and chronic (p ≤ 0.001) treatment with SB-334867 reduced the immobility time (Fig. 3A). The number of crosses in the OFT decreased significantly in CMS treated rats (p ≤ 0.05), which was reversed by chronic SB treatment, as shown in Fig. 3B. Similarly, The ANOVA analysis of the sucrose preference test showed significant differences between groups (F (5, 36) = 6.819, P = 0.0001). The decrease of sucrose consumption from 80.1 ± 3.5% in naïve rats to 67.5 ± 2.7% in CMS-treated animals, was significantly lower according to the Tukey post-hoc test (p ≤ 0.05). Chronic SB treatment reversed the SPT results, as depicted in Fig. 3C (p ≤ 0.05). A reduction in sucrose consumption was also observed in the CSB-only group as compared to the naïve animals Fig. 3C (p ≤ 0.05).

### 3.2 Low gamma band power increased in the CMS model

**Fig. 4:**
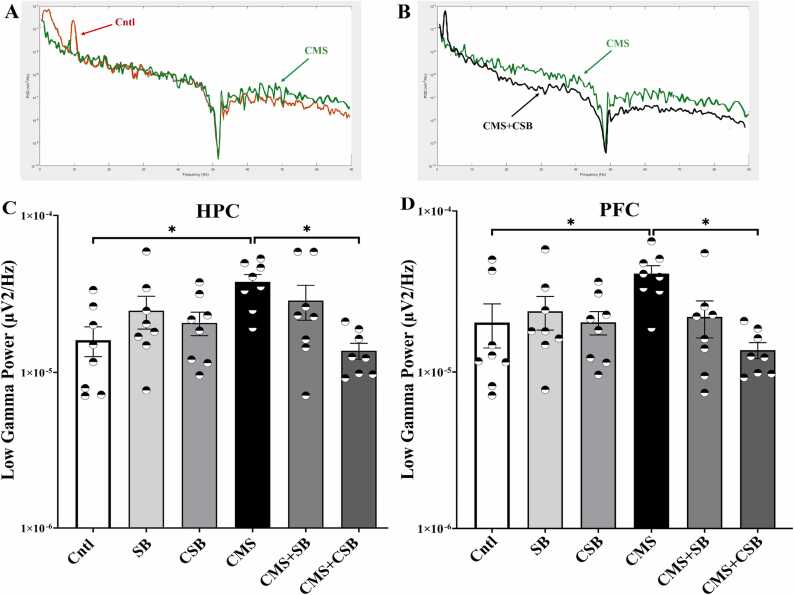
Comparison of sample PSD traces and averaged power in low-gamma band range. Power spectral density diagrams of control and CMS-treated rats (A) and CMS versus CMS+CSB treated ones (B) were compared. Low-Gamma band power in the hippocampus (C) and the prefrontal cortex (D) of different treatment groups. Local field potential recordings were recorded after 3 weeks of intervention. The power of 30–50 Hz frequency band were calculated and depicted as Mean ± SEM. (One-way ANOVA; Bonferroni correction, * p ≤ 0.05, n = 8).

The analysis of local field potential recordings indicated that 3 weeks of exposure to chronic mild stress caused a significant elevation (p ≤ 0.05) in low gamma band power both in the HPC and the PFC. The results of one-way ANOVA in the low-gamma band power (F (5, 42) = 3.610, P = 0.0083) revealed a significant difference between treatment groups. Fig. 4A shows two sample PSD traces of control and CMS rats, while Fig. 4B compares PSD traces of CMS and CMS+CSB treated animals. The low gamma power in the HPC rose from 2E-05 ± 5E-06 µV^2^/Hz of control rats to 4.1E-05 ± 4E-06 µV^2^/Hz in CMS treated animals (Fig. 4C), and from 1.5E-05 ± 3E-06–3.6E-05 ± 3E-06 in the PFC (Fig. 4D).

### 3.3 Chronic SB-334867 injection suppressed the low gamma band power in the CMS model

Chronic administration of SB-334867 reduced the low gamma power from 4.1E-05 ± 4E-06 µV^2^/Hz in the model rats to 1.3E-05 ± 1.3E-06 µV^2^/Hz in CMS+CSB treated ones in the HPC (Fig. 4C) and from 3.6E-05 ± 3E-06 µV^2^/Hz in CMS rats to 1.3E-05 ± 1.4E-06 µV^2^/Hz in the PFC (Fig. 4D), which was statistically significant (p ≤ 0.05).

### 3.4 Theta and high gamma oscillations were also affected by SB-334867 treatment

**Fig. 5:**
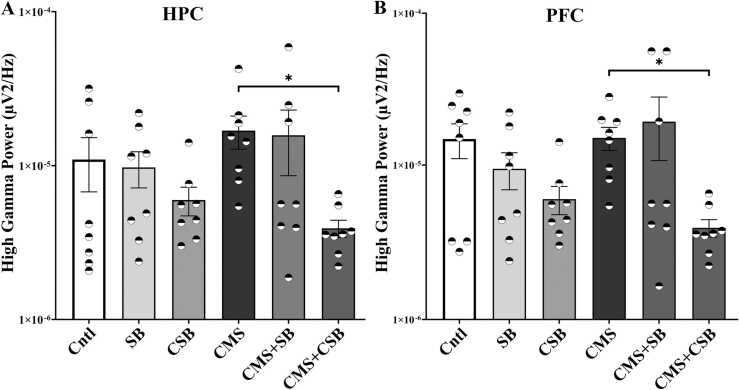
High-Gamma band power in the hippocampus (A) and the prefrontal cortex (B) of different treatment groups. The power of 50–80 Hz frequency band were calculated and illustrated as Mean ± SEM. (One-way ANOVA; Bonferroni correction, * p ≤ 0.05, n = 8).

**Fig. 6:**
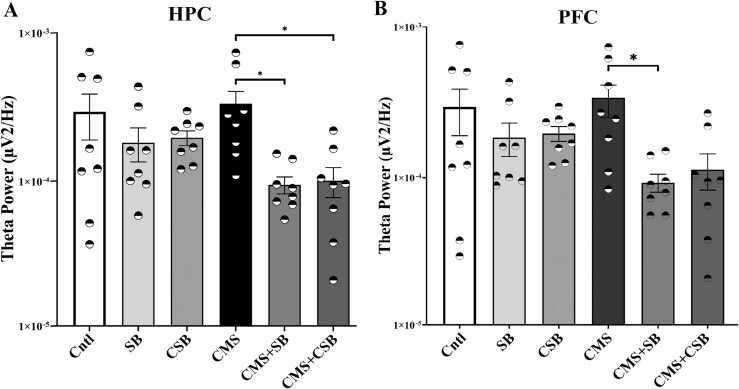
Theta band power alterations in the hippocampus (A) and the prefrontal cortex (B). The power of 4–8 Hz frequency range were analyzed in MATLAB software and depicted as Mean ± SEM. (One-way ANOVA; Bonferroni correction; * p ≤ 0.05, n = 8).

The results of one-way ANOVA in the theta (F (5, 42) = 2.562, P = 0.0413) and high-gamma band power (F (5, 42) = 2.103, P = 0.044) revealed a significant difference between treatment groups. Chronic mild stress did not alter the power of other band frequencies, but chronic SB treatment decreased high gamma power (p ≤ 0.05) in the HPC and the PFC regions (Fig. 5A and B, respectively). As shown in Fig. 6A, in the HPC theta power was suppressed by both acute and chronic SB treatment, while the theta power in the PFC was impacted just by acute SB (Fig. 6B).

### 3.5 HPC-PFC coherence decreased in delta, theta, and alpha band power of CMS-treated rats

**Fig. 7:**
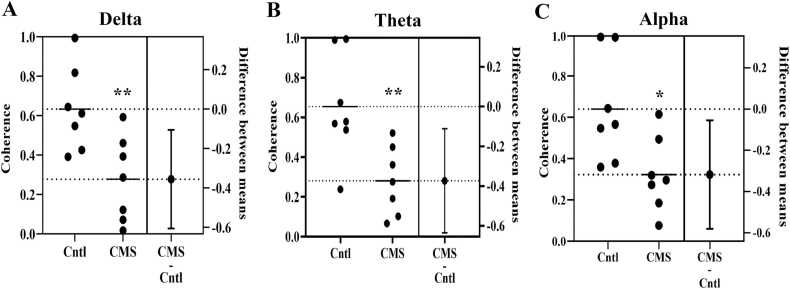
The coherence in the hippocampal-prefrontal cortex interactions decreased in the delta (A), theta (B), and alpha (C) band frequencies of the rat model of depression. The interplay of the hippocampal and prefrontal field potential oscillations between control and CMS-treated rats are shown as Mean ± SEM. (independent t-test, * p ≤ 0.05, ** p ≤ 0.01, n = 8).

As depicted in Fig. 7a significant alteration in the HPC-PFC coherence was observed in the delta (F (3, 51) = 3.485, P = 0.0223), theta (F (3, 48) = 3.758, P = 0.0167), and alpha (F (3, 48) = 1.767, P = 0.0166) band frequencies, between different treatment groups. According to the post-hoc analysis, the HPC-PFC coherence decreased from 0.63 ± 0.07 in healthy rats to 0.28 ± 0.07 in CMS treated animals, in the delta band range (p ≤ 0.01). While, a reduction from 0.65 ± 0.09–0.28 ± 0.06 and from 0.64 ± 0.09–0.32 ± 0.06 was observed in the theta (p ≤ 0.01) and alpha (p ≤ 0.05) band range, respectively.

## 4 Discussion

The results of this study demonstrated that chronic mild stress can boost the low-gamma frequency band's power in the hippocampus and prefrontal cortex. Even though the chronic mild stress model was unable to cause noticeable changes in other frequency bands, SB-334867, a selective antagonist of orexin-1 receptors, was able to reduce the power of the low and high-gamma frequency bands in the hippocampus and the prefrontal cortex over the course of two weeks. Moreover, both acute and chronic injection of SB reduced the theta band power in the hippocampus area compared to CMS-treated animals. In the prefrontal cortex, only acute inhibition of orexin 1 receptors (acute SB administration in CMS-treated rats) was able to diminish the theta band power in comparison to the CMS group. These outcomes are consistent with the earlier study's finding that long-term use of SB-334867 reduced the behavioral symptoms associated with depression (Mirbolouk, 2023). Surprisingly, CSB treatment deceased the sucrose consumption in intact rats, while in CMS+CSB the sucrose preference increased. This finding suggests that the orexin system may be dysregulated in depression, leading to an overactivation of the orexin-1 receptor, and antagonizing it helps to restore balance and alleviate depressive-like behaviors. While, in naïve rats, antagonizing the orexin-1 receptor may disrupt normal brain function and lead to a decrease in sucrose preference. Thus, the effects of CSB may be context-dependent, and further research is needed to understand the underlying mechanisms.

The increased low gamma band power observed in the HPC and PFC of CMS-treated rats may be indicative of hyperarousal and stress-induced neuronal activation (Guan, 2022). Attention, memory, and cognitive processing are linked to low gamma oscillations, which may be influenced by orexinergic inputs originating from the lateral hypothalamus (Rasmussen, 2007). CMS may boost orexin receptor signaling and raise orexin levels, which would enhance low gamma activity in the HPC and PFC (Łupina, 2018). By blocking the orexinergic stimulation of low gamma oscillations in the HPC and PFC, the orexin-1 receptor antagonist SB-334867 may lessen the hyperarousal and neuronal activation driven by stress. Additionally, SB-334867 may restore the excitatory-inhibitory neurotransmission balance in certain brain regions, which CMS may have disturbed. The decrease in theta and high gamma power in the HPC and PFC by SB-334867 may indicate a reduction in hippocampal-prefrontal synchronization, which are involved in learning, memory, and executive functions Domanski et al. (2023). SB-334867 may impair the orexin-mediated modulation of theta and high gamma rhythms, which may depend on the dose, duration, and timing of the drug administration.

Theriault et al., reported alterations in gamma band power after stress exposure; according to their study stress-resilient females displayed higher dorsal hippocampal theta power and cortical gamma power, while stress-resilient males displayed an increase in high gamma coherence (Thériault, 2021). Similarly, it was reported that mouse model of chronic restraint stress also exhibits gamma oscillation dynamics during the treatment procedure of depressive-like behaviors (Khalid, 2016). Gazit T. et al., reported a decrease in the gamma band power in the ventral tegmental area (VTA) of the Flinders Sensitive Line model for depression, which was reversed after programmed deep brain stimulation in the VTA (Gazit, 2015). In other experiments, optogenetic stimulation of gamma oscillations has been used to manipulate mood-related behavior in mice. For example, one study found that optogenetic stimulation of gamma oscillations in the prefrontal cortex was able to reduce the symptoms of depression in a mouse model of the disorder (Fitzgerald, 2018).

In a study conducted on rats with a genetic model of depression, the recording of field potentials indicates a decrease in low gamma frequency oscillations in the vmPFC and NAc region (Hajós, 2003). Likewise, an EEG study on people suffering from depression shows a decrease in the gamma rhythm in resting state in the anterior cingulate cortex (Pizzagalli, 2006). It has also been reported that systemic prescription of noradrenergic antidepressants such as reboxetine increases the gamma band power. In our electrophysiological findings, CMS-treated rats showed an increase in the power of the low gamma band in the hippocampus and prefrontal cortex, which was significantly reduced by the chronic administration of the orexin antagonist.

On the other hand, it has been reported that antidepressants that boost the serotoninergic system, such as citalopram and fluoxetine, reduce the gamma band power in rats. Also, the release of serotonin through the electrical stimulation of the dorsal raphe nuclei also leads to a decrease in the gamma band power in animals Akhmetshina et al., (2016);. These reports are consistent with the results of the present study, as the gamma band power in the HPC and the PFC was reduced by the anti-depressant effect of SB-334867.

The origin of the frontal theta rhythm is in the area of the anterior cingulate cortex (ACC), which is an important area of the brain in controlling behavior and emotions. Also, the dorsal ACC is a fundamental area in the neurobiology of depressive disorders (Ishii, 2014). Depression causes various theta band abnormalities. The majority of research reveal that depressed people have higher theta ACC activity. Since this area's activity is engaged in resolving emotional conflicts, depression's high levels of activity in this region are a reflection of the fronto-cingulate neural network's forced activity, which modifies and controls the emotional state (Pizzagalli, 2011). Moreover, some studies indicate that theta activity declines in depression, which may be related to a decline in activity in the networks that process emotions Coutin-Churchman and Moreno (2008). Despite the fact that in animal model theta bands did not change much as the result of this investigation, sad animals treated with SB had lower theta band power in both the HPC and PFC regions.

Reduced functional connectivity between the hippocampus and the ventral and posterolateral prefrontal cortex contributes to emotional and cognitive impairment in depressive individuals, based on research done on depressed patients in resting state. Also, they demonstrated a strong link between healthy fornix white matter and a proper functional connectivity in the prefrontal - hippocampal circuit in healthy individuals (Geng, 2016). In conjunction with the synaptic transmissions of this circuit and the significant inputs from the ventral hippocampal region (vCA1), heightened rhythmic synchronization between the hippocampus and the mPFC has been experimentally demonstrated in cognitive functions (Adhikari et al., (2010).

A limitation of the present study was that we evaluated the alterations in the canonical band power in different groups; it should be considered that according to Donoghue et al., altered oscillations in narrow-band power may be due to changes in periodic components (like power, shift in the center frequency, or even changes in broadband power) or it can be a result of variations in aperiodic (1/f-like) components of the signal, which can lead to a misinterpretation of the results Donoghue et al., (2020). On the other hand, we assessed the alterations in local field oscillations of CMS treated rats in the anesthetized condition, which could affect the spectral power. Hanrahan et al. reported that in human propofol-induced anesthesia can lead to the generation of large-amplitude spike-like LFP activity, decreased AP firing rates and diminish high-frequency power in LFP spectra (Hanrahan, 2013). Ma et al. reported that application of ketamine in rhesus macaque monkeys beside the impairment of working-memory, can enhance the low-gamma-band power and dampen the beta-band activities in the lateral prefrontal cortex (Ma, 2018). To the best of our knowledge urethan is reported to induce neocortical slow oscillation and suppress hippocampal neuronal synchronization (Yagishita, 2020). Nevertheless, it should be noted that in this study, all the recordings were made under the same conditions and the alterations observed in field potential oscillations are contrasted amongst various groups in a unique setting. Therefore, the changes in spectral power and other criteria can be attributed to the effects exerted by chronic stress and SB treatment. It is reported that urethane anesthesia induces a novel brain state of sustained slow oscillations (0.5–1.5 Hz) associated with a short-term synaptic plasticity deficit at the subiculum-mPFC pathway (Kiss, 2011). Also, two distinct alternating oscillatory patterns were observed: (1) high amplitudes and slow rhythms in the LFP, similar to slow wave sleep patterns; and (2) an increase in faster frequency band power (>4 Hz), similar to REM oscillatory activity (Lopes-Aguair, 2020). Considering that LFP recordings in different groups were performed under the same conditions, we suppose that these findings do not interfere with the results of the present study. The current study only included male rats; more research is required to evaluate the differences in female animals given the gender disparities in the pathophysiology of depressive-like behaviors. The findings of this study demonstrated that CMS treated animals have lower levels of delta, theta, and alpha band coherence between the hippocampus and the prefrontal cortex. This outcome is consistent with earlier publications, which found that theta band coherence, particularly in the frontal region, is associated with the degree of depression and may serve as a marker for depression (Lee, 2011).

## 5 Conclusion

In CMS-treated animals, the power of the low-gamma frequency band increases in the hippocampus and prefrontal cortex, which then declines again with chronic orexin-1 receptor inhibition. Moreover, SB therapy for these animals can boost the strength of high theta and gamma frequency bands. The coherence between the hippocampus and the PFC is also diminished in the low-frequency bands of delta, theta, and alpha in the CMS model animals.

## Ethical Approval statement

Effort was made to reduce the number of animals utilized and their suffering. The "Ethical Committee of the Faculty of Medicine" established ethical standards for all research, and these standards were followed religiously, drawing exclusively from the "NIH Guide for the Care and Use of Laboratory Animals." The Research Ethics Committee at Guilan University of Medical Sciences gave this study ethical approval (IR.GUMS.REC.1398.509).

## CRediT authorship contribution statement

Conception and design of study: **K. Rohampour, B. Khakpour, M. Rostampour**; Acquisition of data: **B. Mirbolouk, A. Jafari, B. Khakpour**; Analysis and/or interpretation of data: **B. Khakpour, K. Rohampour, M. Rostampour**; Writing – original draft. **B. Mirbolouk, K. Rohampour**; Writing – review & editing: **K. Rohampour, B. Khakpour, M. Rostampour, A. Jafari, B. Mirbolouk.**

## Declaration of Competing Interest

There is no conflict of interest for any of the contributing authors. The authors alone are responsible for the content and writing of the paper.
